# Dynamic susceptibility contrast ^19^F‐MRI of inhaled perfluoropropane: a novel approach to combined pulmonary ventilation and perfusion imaging

**DOI:** 10.1002/mrm.27933

**Published:** 2019-08-29

**Authors:** Mary A. Neal, Benjamin J. Pippard, A. John Simpson, Peter E. Thelwall

**Affiliations:** ^1^ Institute of Cellular Medicine Newcastle University Newcastle upon Tyne United Kingdom; ^2^ Newcastle Magnetic Resonance Centre Newcastle University Newcastle upon Tyne United Kingdom; ^3^ Respiratory Medicine Newcastle upon Tyne Hospitals NHS Foundation Trust Newcastle upon Tyne United Kingdom

**Keywords:** ^19^F‐MRI, dynamic susceptibility contrast, lung, perfluoropropane, perfusion, ventilation

## Abstract

**Purpose:**

To assess alveolar perfusion by applying dynamic susceptibility contrast MRI to ^19^F‐MRI of inhaled perfluoropropane (PFP). We hypothesized that passage of gadolinium‐based contrast agent (GBCA) through the pulmonary microvasculature would reduce magnetic susceptibility differences between water and gas components of the lung, elevating the $T_{2}^{*}$ of PFP.

**Methods:**

Lung‐representative phantoms were constructed of aqueous PFP‐filled foams to characterize the impact of aqueous/gas phase magnetic susceptibility differences on PFP $T_{2}^{*}$. Aqueous phase magnetic susceptibility was modulated by addition of different concentrations of GBCA. In vivo studies were performed to measure the impact of intravenously administered GBCA on the $T_{2}^{*}$ of inhaled PFP in mice (7.0 Tesla) and in healthy volunteers (3.0 Tesla).

**Results:**

Perfluoropropane $T_{2}^{*}$ was sensitive to modulation of magnetic susceptibility difference between gas and water components of the lung, both in phantom models and in vivo. Negation of aqueous/gas phase magnetic susceptibility difference was achieved in lung‐representative phantoms and in mice, resulting in a ~2 to 3× elevation in PFP $T_{2}^{*}$ (3.7 to 8.5 ms and 0.7 to 2.6 ms, respectively). Human studies demonstrated a transient elevation of inhaled PFP $T_{2}^{*}$ (1.50 to 1.64 ms) during passage of GBCA bolus through the lung circulation, demonstrating sensitivity to lung perfusion.

**Conclusion:**

We demonstrate indirect detection of a GBCA in the pulmonary microvasculature via changes to the $T_{2}^{*}$ of gas phase PFP within directly adjacent alveoli. This approach holds potential for assessing alveolar perfusion by dynamic susceptibility contrast ^19^F‐MRI of inhaled PFP, with concurrent assessment of lung ventilation properties, relevant to lung physiology and disease.

## 1 INTRODUCTION

The lungs present challenges for diagnostic MRI due to low tissue water content, respiratory and cardiac motion, and magnetic field inhomogeneity arising from differences in magnetic susceptibility at ubiquitous air–tissue interfaces.^1^ The development of hyperpolarized ^3^He and ^129^Xe gas MRI has addressed some of these challenges to provide quantitative assessment of regional gas distribution and wash‐out dynamics in a variety of pulmonary disorders.^2^, ^3^, ^4^, ^5^ However, the requirement for polarizing equipment and expertise has largely restricted this technique to research settings and specialist centers. An alternative approach involving ^19^F‐MRI of inhaled perfluoropropane (PFP) has shown potential for assessing ventilation properties in healthy volunteers and patients with respiratory disease,^6^, ^7^, ^8^, ^9^ building on extensive preclinical^10^, ^11^, ^12^, ^13^, ^14^ and ex vivo^15^ studies. PFP has a short in vivo T_1_ (approximately 12 ms at 3.0 Telsa [T]^7^) that enables a short TR and high degree of signal averaging. This, in concert with its 6 magnetically equivalent ^19^F nuclei per molecule and inert nature (permitting inhalation at high concentrations), facilitates in vivo imaging of PFP at thermal polarization, avoiding the need for hyperpolarization with a resultant technical simplicity that offers potential for clinical application.

The PFP molecule is substantially larger than ^3^He and ^129^Xe atoms and has a significantly slower diffusion coefficient (0.07 cm^2^/s,^16^ compared to diffusion coefficients in air of 0.88 cm^2^/s^17^ and 0.14 cm^2^/s^18^ for ^3^He and ^129^Xe, respectively). Its fluorine resonances exhibit a short in vivo $T_{2}^{*}$ (approximately 2 ms on inspiration at 3.0 T,^7^ compared to 14 ms^19^ and 24 ms^20^ for ^3^He and ^129^Xe, respectively) with limited motional narrowing compared to ^3^He and ^129^Xe, where faster diffusion through alveolar magnetic susceptibility gradients limits shortening of $T_{2}^{*}$. The resultant sensitivity of PFP $T_{2}^{*}$ to the lung magnetic microenvironment presents some technical challenges for MRI of inhaled PFP; however, it also provides opportunities to report on lung tissue functional properties that include monitoring lung perfusion via alteration of pulmonary magnetic susceptibility gradients. Modulation of tissue magnetic susceptibility through the introduction of a gadolinium‐based contrast agent (GBCA), with consequent change in $T_{2}^{*}$, forms the basis of dynamic susceptibility contrast (DSC) MRI. This well‐established technique has been used most widely in the assessment of brain perfusion where the paramagnetic properties of intravenously administered GBCA cause increased heterogeneity of tissue magnetic susceptibility in perfused regions, reducing the $T_{2}^{*}$ of tissue water.^21^, ^22^, ^23^

We hypothesized that the magnetic field heterogeneity of the lung could be reduced by the presence of a paramagnetic GBCA in the pulmonary circulation, bringing the magnetic susceptibilities of aqueous and gas components of the lung closer together as the paramagnetic nature of gadolinium counters the diamagnetic properties of tissue water. Specifically, we tested whether contrast agent bolus passage through the lung microvasculature would result in a transient elevation of the $T_{2}^{*}$ of inhaled PFP, utilizing lung‐representative phantoms, preclinical models, and subsequent demonstration of feasibility in healthy human volunteers. Our approach builds on previous preclinical demonstrations of reducing lung magnetic susceptibility gradients using paramagnetic and superparamagnetic contrast agents with hyperpolarized ^3^He‐MRI^24^, ^25^ by employing PFP as a gas phase imaging agent to perform dynamic measurement of susceptibility contrast and by translating this approach to humans.

## 2 METHODS

### 2.1 Phantom study

Lung‐representative phantoms (n = 36) were constructed of stable aqueous foams. The gas phase of the foams contained a 79:21 ratio mixture of PFP and oxygen, and the aqueous phase comprised an aqueous ovalbumin solution (1:6 weight/weight ratio of pasteurized egg white powder (Dr. Oetker Ltd., UK) and water). Foam was produced by repeatedly passing gas and aqueous components between a pair of syringes through a 2 mm diameter restriction until a stable and homogenous foam was formed (typically achieved after 20 passages). Foam volume was 15 mL for all samples. Digital photomicrographs were acquired with a microscope (Celestron LLC, Torrance, CA) from a sample of each of the foams placed on a microscope slide immediately prior to MR acquisitions. Mean bubble diameter was measured manually from photomicrographs using ImageJ^26^ from approximately 100 bubbles within the FOV of each sample image. The magnetic susceptibility of the aqueous component was altered by the addition of a GBCA (Gadobutrol, Bayer Schering Pharma, UK) at 12 different concentrations over the range 0 to 70 mM, with 3 phantoms prepared at each concentration. Three gas‐only samples (i.e., containing the PFP/oxygen gas mixture alone, with no aqueous component) were also prepared for comparison. ^19^F‐MR data were acquired from foam phantoms and gas‐only samples with a Philips Achieva 3.0 T scanner (Philips, Best, The Netherlands) equipped with a custom 25 mm diameter 6‐turn solenoid ^19^F coil, using a pulse‐acquire sequence (TR = 34 ms; flip angle = 90°; acquisition bandwidth (BW) = 8 kHz; number of datapoints = 256; number of averages = 10). The $T_{2}^{*}$ of PFP was determined by fitting Equation 1 to the magnitude of the ^19^F pulse‐acquire free induction decay, where the equation describes monoexponential decay of the ^19^F signal plus the mean magnitude of baseline noise in the FID.

(1) $$S=\left(S_{0}\times e^{-t/T_{2}^{*}}\right)+c.$$

A simple model was used to describe the relationship between difference in magnetic susceptibility between gas and aqueous components of the foam and the resultant change in $T_{2}^{*}$. The magnetic susceptibility of the aqueous phase of the foam was defined as the sum of magnetic susceptibilities of water and GBCA components. The absolute value of the difference in magnetic susceptibility between gas and aqueous components, Δχ, was calculated using Equation 2, where χ^gas^ = 0.4 ppm, χ^aq^ = −9.05 ppm,^27^ the molar magnetic susceptibility of GBCA (χ_m_^Gd^) = 320 ppm L mol^−1^,^28^ and C_GBCA_ is the concentration of GBCA in the aqueous component of the foam.

(2) $$\Delta \chi =\left|\left(\chi^{\mathrm{gas}}-\left(\chi^{\mathrm{aq}}+\left(\chi_{m}^{\mathrm{Gd}}\times C_{\mathrm{GBCA}}\right)\right)\right)\right|.$$

The relationship between Δχ and change in PFP transverse relaxation rate ($\Delta R_{2}^{*}$) was modeled on the relationship observed in and used for analysis of brain DSC ^1^H‐MRI data,^22^ where $\Delta R_{2}^{*}$ is linearly proportional to Δχ resultant from contrast agent administration. Equation 3 describes this relationship, where *k* is a proportionality constant dependent on foam microstructural properties and the diffusion coefficient of PFP that links the magnitude of Δχ to the magnitude of PFP $\Delta R_{2}^{*}$.^21^, ^22^, ^29^ Equation 3 was fitted to PFP $R_{2}^{*}$ measurements made from phantoms.

(3) $$\Delta R_{2}^{*}=k\times \;\Delta \chi .$$

### 2.2 Preclinical study

Preclinical experiments were performed under a project license granted by the UK Home Office in accordance with the Animals (Scientific Procedures) Act 1986. Four male C57BL/6 mice were anaesthetized by intraperitoneal injection of a hypnorm/midazolam/water mixture (1:1:2 volume ratio, dose 10 mL/kg body weight). A tail vein cannula was sited for administration of GBCA (Gadobutrol, Bayer Schering Pharma). A custom breathing mask was positioned over the mouse head, allowing delivery of a 79% PFP/21% oxygen gas mixture (BOC Ltd., Guildford, UK). MR data were acquired using a Varian 7.0 T spectrometer and console (DirectDrive system, Varian, Palo Alto, CA) and a custom 38 mm diameter ^19^F birdcage coil. Respiratory rate and body temperature were monitored throughout the study, with body temperature maintained using an air heater.

Mice inhaled the PFP/oxygen gas mixture for 1 min prior to and during MR scans to measure PFP $T_{2}^{*}$, which comprised a 2D spoiled gradient echo imaging sequence with multiple repetitions at different TEs (flip angle = 90o; BW = 50 kHz; TE = 1.0, 1.2, 1.4, 1.6, 1.8 ms; TR = 4.0 ms; FOV = 50 × 50 mm^2^; acquisition matrix = 64 × 64; number of averages = 200; scan duration = 256 s). The 5 scans with different echo time were acquired in an interleaved manner without respiratory gating such that the resultant relaxation time measurements represent an average over the scan acquisition duration. PFP $T_{2}^{*}$ measurements were performed prior to and at 3.5 min after each of 5 intravenous administrations of Gadobutrol (Bayer Schering Pharma) (1M; 200 μL per administration). The time interval between respective administrations was approximately 5 min (mean ± SD = 307 ± 17 s). Mice were removed from the scanner on completion of MR imaging and euthanized by cervical dislocation while still under anaesthesia.

PFP $T_{2}^{*}$ was calculated from a 4 × 4 mm^2^ region of interest in the center of the left lung of each mouse by fitting a monoexponential function to the decay in ^19^F signal intensity with increasing TE.

### 2.3 Human study

This study was reviewed by the National Health Service National Research Ethics Service and approved by the National Health Service Health Research Authority. Four healthy volunteers (3 males, aged 24, 28, and 34 years; 1 female, aged 28 years) provided written informed consent to participate and were screened for study eligibility, including normal lung function assessed by spirometry. All participants were nonsmokers (2 never‐smokers; 2 previous social smokers, each with < 1 pack years) and in good health, with no history of respiratory, cardiac, or other significant medical problems.

Imaging was performed using a Philips Achieva 3.0 T MRI scanner equipped with a 20 cm diameter ^19^F surface coil (PulseTeq Ltd., Woking, UK) for ^19^F‐MRI acquisitions. A cannula was sited in an antecubital fossa vein for intravenous contrast administration. Participants were positioned supine with the surface coil placed centrally below their upper back such that the top of the coil was aligned with the clavicles. Heart rate and oxygen saturations were monitored throughout via a MR‐compatible pulse oximeter (Nonin 7500FO, Nonin Medical Inc., Plymouth, MA). Scout ^1^H‐MR images (coronal views) were acquired using the scanner's body coil with a multislice 2D gradient echo sequence (flip angle = 10°; TE = 1.8 ms; TR = 4.0 ms; BW = 450 Hz/pixel; FOV = 450 × 450 × 250 mm^3^; acquisition matrix = 192 × 96; scan duration = 26 s).

Participants were instructed to inhale a mixture of 79% PFP/21% oxygen (BOC Ltd, Guildford, UK) from a 25 L reservoir bag via a mouthpiece and 3‐way valve. Each participant performed 3 deep breaths of the gas mixture, followed by a 30 s breath‐hold at maximal inspiration (i.e., total lung capacity). Gadobutrol (Gadovist 1.0 mmol/mL, Bayer Schering Pharma) was administered at a dose of 0.2 mL/kg (0.2 mmol/kg equivalent) according to participant weight via a power injector (rate = 5 mL/s) concurrent with the start of the breath‐hold, followed by a 20 mL saline flush at the same rate. Dynamic ^19^F‐MR data acquisitions commenced immediately prior to the start of the breath‐hold. Breath‐hold compliance was confirmed by participant observation. On completion of the breath‐hold, participants reverted to breathing room air.

For the first participant, a dynamic unlocalized ^19^F pulse‐acquire FID (flip angle = 90°; BW = 8000 Hz; TR = 250 ms; number of datapoints = 256; number of dynamics = 350; scan duration = 88 s) was acquired on 2 occasions during the scan session: once without and once with concurrent administration of GBCA. For the remaining 3 participants, a dynamic ^19^F 2D spoiled gradient echo imaging sequence (flip angle = 50°; TE = 1.7 ms; TR = 4.2 ms; BW = 500 Hz/pixel; FOV = 300 × 300 × 200 mm^3^; acquisition matrix = 24 × 24; number of dynamics = 600; scan duration = 60 s) was acquired. Dynamic ^19^F‐MR spectroscopy data were analyzed by fitting Equation 1 to the amplitude of the ^19^F free induction decay, allowing calculation of PFP $T_{2}^{*}$ for each repetition of the dynamic acquisition. Baseline $R_{2}^{*}$ was calculated from the mean $R_{2}^{*}$ over the first 7 s of the experimental timecourse, and the change in $R_{2}^{*}$ (i.e., $\Delta R_{2}^{*}$) was calculated for each point in the timecourse. Images in the dynamic ^19^F‐MRI dataset were averaged to a temporal resolution of 3 s (i.e., 30 dynamics per average), and the change in ^19^F signal amplitude over the dynamic scan series was calculated on a pixelwise basis relative to baseline signal amplitude (defined as the first 7.5 s of data acquisition, prior to arrival of GBCA in the pulmonary circulation).

## 3 RESULTS

### 3.1 Lung‐representative phantoms

**Figure 1:**
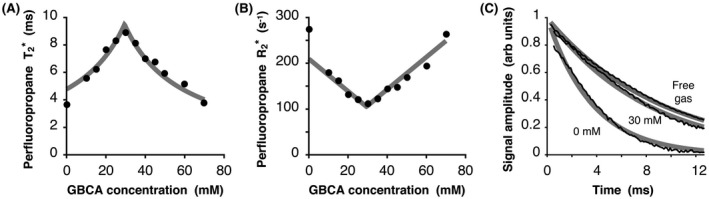
Impact of GBCA on the $T_{2}^{*}$ (A) and $R_{2}^{*}$ (B) of PFP in a lung‐representative phantom (aqueous foam) with increasing GBCA content within the aqueous component of foam samples (•) and model fit to the data (—). The magnetic susceptibilities of aqueous and gas components are matched at a GBCA concentration of 29.3 mM. The transverse relaxation rate of PFP increases linearly with change in GBCA concentration from this susceptibility match point. (C) FID signal amplitudes for a gas‐only sample and for foam samples containing 0 and 30 mM GBCA (black lines), and model fits to the data (gray lines). FID, free induction decay; GBCA, gadolinium‐based contrast agent; PFP, perfluoropropane

PFP foams had a bubble diameter of 29 ± 12 µm (mean ± SD), which was unaffected by the addition of GBCA. PFP‐filled aqueous foams containing no GBCA exhibited a shorter $T_{2}^{*}$ than that of gas‐only samples (3.7 ± 0.1 ms compared to 9.1 ± 0.1 ms, respectively). Figure 1A shows the change in PFP $T_{2}^{*}$ at 3.0 T as GBCA concentration in the aqueous component of the foam was increased. Measured PFP $T_{2}^{*}$ reached a maximum of 8.5 ± 0.2 ms at a GBCA concentration of 29 mM, where the magnetic susceptibility of the aqueous component matches that of the gas component (Δχ = 0). Increasing the concentration of GBCA further caused a reduction in PFP $T_{2}^{*}$ as magnetic susceptibilities of the aqueous and gas components diverged. Figure 1B shows a linear increase in PFP $R_{2}^{*}$ as the absolute value of Δχ increases away from this match point. Solid lines represent a fit of Equations 2 and 3 to the measurements, which yield a calculated $T_{2}^{*}$ of 9.5 ms at Δχ = 0, comparable to the measured $T_{2}^{*}$ of gas‐only samples (9.1 ± 0.1 ms). Whilst it is likely that the presence of ovalbumin in the aqueous component of the foam altered its magnetic susceptibility away from the literature value of χ^aq^ used to fit Equations 2 and 3 to the data, the agreement between inflection points of data and fit suggest that the change in χ^aq^ away from −9.05ppm was minor. 95% confidence intervals for $T_{2}^{*}$ fits calculated for datapoints shown in Figure 1A,B yield error bars within the datapoint markers. Figure 1C shows the magnitude of ^19^F FID signal amplitudes for a gas‐only sample and for foam samples containing 0 and 30 mM GBCA (black lines), as well as fits to the data from which $T_{2}^{*}$ values were determined (gray lines). The plots show $T_{2}^{*}$ relaxation exhibiting slight deviation from exponential decay.

### 3.2 Preclinical study

**Figure 2:**
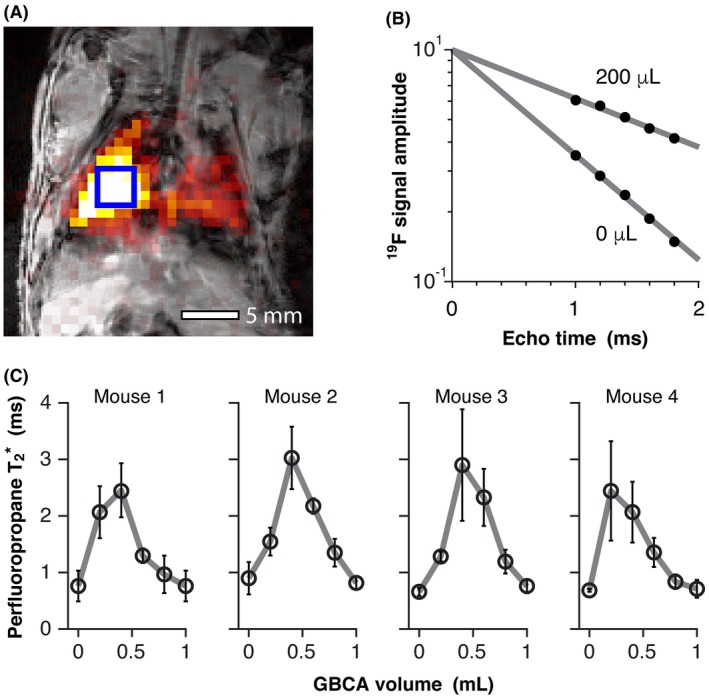
(A) Representative gradient echo ^19^F‐MRI image of inhaled PFP acquired from a mouse (colormap), overlaid on a ^1^H‐MRI anatomical image (grayscale). Signal amplitude measurements made from lung regions of interest (example shown in blue) were used for calculation of $T_{2}^{*}$ values. (B) Change in PFP signal amplitude with increasing TE (•) measured from a lung region of interest in a mouse (mouse 1) prior to and after administration of 200 µL of contrast agent. (C) Impact of intravenous GBCA administration on the $T_{2}^{*}$ of inhaled PFP in 4 mice. Error bars show 95% confidence intervals of exponential fits to the data. $T_{2}^{*}$ is maximal when the magnetic susceptibilities of lung water and gas components are matched, following administration and equilibration of approximately 400 µL of contrast agent

Figure 2A shows a representative gradient echo ^19^F‐MR image acquired from a mouse, overlaid on a ^1^H anatomical scan. A region of analysis for ^19^F signal amplitude determination is shown in blue. Figure 2B shows change in ^19^F signal amplitude with TE measured in a mouse (mouse 1) prior to and after administration of 400 µL of GBCA, illustrating the resultant change in PFP $T_{2}^{*}$. Figure 2C shows the change in in vivo $T_{2}^{*}$ of inhaled PFP in mouse lung (n = 4) at 7.0 T following successive administrations of GBCA. As with lung‐representative phantoms, an increase in PFP $T_{2}^{*}$ is observed from an initial value of 0.7 ± 0.1 ms (mean ± SD) to a maximum value 2.6 ± 0.6 ms following administration of a total of 400 µL of contrast agent. A decrease in PFP $T_{2}^{*}$ was observed with further GBCA administration as magnetic susceptibilities of lung tissue and gas components diverge. Slight differences in the volume of GBCA required for maximal impact on PFP $T_{2}^{*}$ were observed between mice. Body weight varied slightly between animals (30.0, 29.3, 32.1, and 27.7 g for mice 1 to 4, respectively), which may have contributed to this difference.

### 3.3 Human study

PFP gas inhalation and intravenous GBCA administration were well tolerated by all participants, with no adverse events. The mean volume of Gadobutrol administered was 14.1 mL (range 11.6 to 17.0 mL), determined by participant weight (range 58 to 84 kg) and dose (0.2 mL/kg).

**Figure 3:**
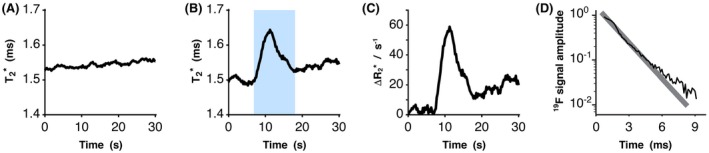
Dynamic measurement of the $T_{2}^{*}$ of inhaled PFP in a healthy participant during a 30 s breath‐hold without (A) and with (B) concurrent intravenous administration of GBCA. PFP $T_{2}^{*}$ is transiently elevated as the GBCA bolus passes through the pulmonary vasculature (highlighted region). (C) Change in PFP $R_{2}^{*}$ over the experimental time‐course, calculated relative to baseline $R_{2}^{*}$ prior to GBCA bolus arrival. (D) Plot of FID signal magnitude (black line) and an exponential fit to the data (gray line) from a single dynamic of the ^19^F spectroscopy series

Figure 3A shows the in vivo $T_{2}^{*}$ of inhaled PFP during a breath‐hold without GBCA administration, measured by ^19^F‐MR spectroscopy in 1 participant. PFP $T_{2}^{*}$ was 1.54 ± 0.05 ms (mean ± SD) over the 30 s breath‐hold. Figure 3B shows PFP $T_{2}^{*}$ in the same participant prior to, during, and following GBCA bolus passage through the pulmonary vasculature. Baseline $T_{2}^{*}$ prior to GBCA bolus arrival was 1.50 ± 0.01 ms. Maximal $T_{2}^{*}$ measured during GBCA bolus passage through the lungs was 1.64 ms, occurring at 11.25 s. The pre‐GBCA $T_{2}^{*}$ seen in Figure 3B differs slightly from the $T_{2}^{*}$ seen in Figure 3A. We attribute this to slight differences in lung inflation level between the 2 scans. A subtle increase in PFP $T_{2}^{*}$ was observed over the breath‐hold duration in Figure 3A. Whilst we have not investigated the origins of this change, one hypothesis would be that this occurs as a result of progressive deoxygenation of the lung gas phase during the breath‐hold. Pulse oximetry measurements showed no change in blood oxygen saturation over the experiment.

Figure 3C shows change in PFP $R_{2}^{*}$ over the experimental time‐course, calculated by comparison to $R_{2}^{*}$ at baseline, showing an elevation in $R_{2}^{*}$ during GBCA bolus passage though the lungs. Figure 3D plots the ^19^F FID signal magnitude of a single dynamic from the ^19^F spectroscopy series (black line). The fit of a decaying exponential function to these data is shown by the gray line from which measured $T_{2}^{*}$ was determined. As with foam samples, slight deviation from the exponential decay was observed.

**Figure 4:**
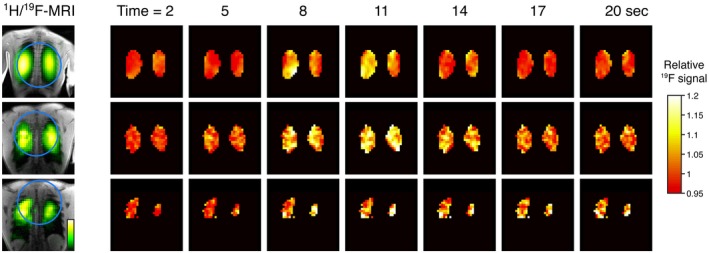
Dynamic ^19^F‐MRI of inhaled PFP acquired before, during, and after passage of a GBCA bolus through the pulmonary vasculature in 3 healthy volunteers. $T_{2}^{*}$‐weighted ^19^F‐MRI images overlaid on ^1^H anatomical scans (left) show blue circles to indicate position of the ^19^F surface coil. Dynamic ^19^F‐MRI data (right) show relative change in $T_{2}^{*}$‐weighted ^19^F‐MRI signal compared to baseline amplitude (i.e., pre‐GBCA bolus arrival). A transient elevation in PFP signal amplitude is observed at approximately 8 to 14 s following GBCA administration, reflecting partial susceptibility matching of lung water and gas components

**Figure 5:**
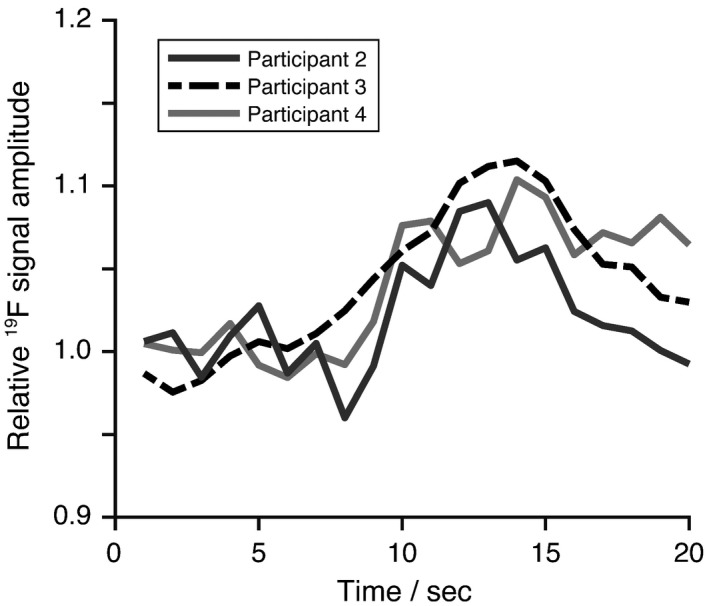
Amplitude of lung ^19^F signal from dynamic $T_{2}^{*}$‐weighted ^19^F‐MRI scans acquired from participants 2, 3, and 4. An elevation in PFP signal amplitude is observed at approximately 8 to 14 s following GBCA administration, reflecting partial susceptibility matching of lung water and gas components

Figure 4A shows baseline $T_{2}^{*}$‐weighted ^19^F‐MR scans (i.e., prior to arrival of the GBCA bolus, generated by averaging the initial 75 dynamics of the ^19^F scan series) overlaid on anatomical ^1^H scans for each of the remaining 3 participants. Positioning of the ^19^F surface coil is represented by blue circles. Figure 4B shows the relative change in PFP signal intensity calculated from dynamic $T_{2}^{*}$‐weighted ^19^F‐MRI scans following GBCA administration in each participant. A transient increase in PFP signal intensity of 8.7% ± 2.1% was observed at approximately 8 to 14 s following injection of Gadobutrol. Data from participant 4 has lower ^19^F signal to noise ratio than data from participants 2 and 3 as a result of poor coil positioning. Plots of ^19^F signal amplitude over the experimental timecourse for each participant are shown in Figure 5.

## 4 DISCUSSION

We have demonstrated a method for assessing lung perfusion properties by employing an intravenously administered GBCA to alter the magnetic susceptibility of pulmonary blood directly adjacent to PFP gas within the alveoli. To our knowledge, this represents the first *in man* demonstration of DSC ^19^F‐MRI assessment of pulmonary perfusion using an approach that is well suited to concurrent ^19^F‐MRI of lung ventilation within a short breath‐hold duration.

Previous ^19^F‐MRI studies have reported the ability to assess ventilation properties using inert fluorinated gases in humans^6^, ^7^, ^8^ and in preclinical^10^, ^11^, ^12^, ^13^, ^14^ and ex vivo models,^15^ demonstrating technical feasibility and enabling characterization of the physical and MR properties of in vivo fluorocarbon gases.^30^, ^31^ The signal‐to‐noise ratio and resultant image quality of ^19^F‐MRI of inhaled PFP is inherently lower than MRI of hyperpolarized ^3^He or ^129^Xe due to its thermal polarization. However, the avoidance of hyperpolarization facilitates dynamic imaging because PFP signal intensity is determined solely by gas concentration and relaxation properties and without confounding factors arising from T_1_‐ and excitation‐mediated loss of hyperpolarization between scans in a dynamic series.

Our studies demonstrate sensitivity of ^19^F‐MRI to pulmonary perfusion by DSC‐MRI. However, unlike the diminishing effect on $T_{2}^{*}$ that is observed in conventional ^1^H DSC‐MRI of the brain,^21^ DSC ^19^F‐MRI causes an elevation of PFP $T_{2}^{*}$ during GBCA bolus passage. This positive contrast is a direct consequence of the paramagnetic GBCA reducing lung Δχ between adjacent gas and tissue components within the lung, an effect that has been reported in previous preclinical studies. Vignaud et al^24^ demonstrated a 3‐fold elevation of the $T_{2}^{*}$ of inhaled hyperpolarized ^3^He following intravenous administration of superparamagnetic iron oxide contrast agent in rats. Similarly, Dimitrov et al^25^ demonstrated partial susceptibility matching of water and gas components in in vivo pig lungs following administration of a GBCA, observed in the phase shift of ^3^He gradient echo images. However, neither study performed dynamic imaging to assess lung perfusion. Vignaud et al. employed imaging once the contrast agent was equilibrated throughout body tissues, whereas Dimitrov et al. performed separate ^3^He inhalations for pre‐ and post‐GBCA imaging, confounding a direct comparison and thus quantitation of lung perfusion properties. Nonetheless, both studies showed that partial or complete susceptibility matching can be achieved in the lungs. Our study data are consistent with these previous findings and extend this concept to human imaging using a DSC approach, drawing on the high sensitivity of PFP $T_{2}^{*}$ to lung microstructural properties. Additionally, our studies in lung‐representative phantoms demonstrate the linear relationship between contrast agent concentration and PFP $R_{2}^{*}$ in situations in which tissue microstructure is constant (i.e., the value of *k* in Equation 3 remains unchanged). This permits quantitation of relative contrast agent content in the lung‐representative phantoms. Whilst *k* may reasonably be expected to remain constant over the breath‐hold duration in our human studies, it remains unknown and is dependent on tissue microstructural properties. Thus, it is possible that *k* may vary across the lung and indeed with underlying pathology, which may confound quantitation of relative contrast agent concentration in vivo. Investigating the relationship between tissue microstructure and *k* may provide an opportunity to extend analysis to a more quantitative approach in the future in vitro and in vivo studies. Applying the approach to the study of respiratory disease, for example in patients with perfusion defects, would be of value to assess the impact of pathology on the magnitude of change in contrast agent concentration over time as well as on the timing of bolus arrival relative to administration within the microvasculature adjacent to alveolar gas.

The baseline PFP $T_{2}^{*}$ values measured in our preclinical studies were shorter than the $T_{2}^{*}$ values obtained from our PFP foam samples and human studies. This reflects the higher field strength used (7.0 T for preclinical studies; 3.0 T for foam and human studies), with greater resultant magnetic susceptibility‐induced field gradients within the lung parenchyma at higher field strength. ^19^F‐MRI of inhaled PFP has been successfully applied at 1.5 T, where PFP $T_{2}^{*}$ is longer due to weaker field gradients within the lung.^8^ The slower $T_{2}^{*}$ relaxation may confer an advantage for conventional and DSC ^19^F‐MRI of inhaled PFP alongside the advantages of lower RF power deposition (specific absorption rate, SAR) in these high flip angle, short TR protocols. However, lower field also confers lower thermal polarization and thus lower signal amplitude. A comprehensive comparison of inhaled PFP ^19^F‐MRI performance at 1.5 T and 3.0 T would be of value in determining optimal MR scanner hardware configuration for this application.

Lung inflation level is known to impact the $T_{2}^{*}$ of inhaled PFP.^32^ Participants in our human studies were instructed to perform breath‐holds at full inspiration during ^19^F‐MRI data acquisition to prevent changes in inflation level confounding the measured effects of GBCA on PFP $T_{2}^{*}$. The data in Figure 3B show slightly higher $T_{2}^{*}$ after GBCA bolus passage through the lung vasculature compared to the pre‐GBCA baseline $T_{2}^{*}$. This may arise from extravasation of GBCA from the pulmonary vasculature during bolus passage. PFP $T_{2}^{*}$ relaxation deviated from monoexponential decay in both foam and in vivo studies, evident in the early timepoints of the FID (0 to 3 ms) shown in Figure 3D. This deviation is similar to that observed in ^3^He studies of rat lung following intravenous administration of iron oxide nanoparticles to alter aqueous phase magnetic susceptibility.^24^ This effect has been attributed to the structural complexity of lung tissue, where magnetic susceptibility gradients occur over a range of length scales and across a range of diffusion restriction dimensions. Slight deviation from monoexponential decay was also observed in the gas‐only sample. Imperfect B_0_ homogeneity may have contributed to nonmonoexponential $T_{2}^{*}$ decay in this sample as well as in foam samples and human studies.

The use of a ^19^F surface coil for detection of inhaled PFP in our human studies prevented imaging of the entire lung volume but was nonetheless sufficient to demonstrate technical feasibility of our approach. Previous studies have demonstrated visualization of the entire lung volume with inhaled PFP.^6^, ^7^, ^8^ Further optimization of ^19^F scan performance through the use of multichannel RF coil arrays,^8^, ^33^ as well as scan protocol acceleration with compressed sensing,^34^ offer potential to implement dynamic 3D ^19^F‐MRI. This has clear implications for performing concurrent whole‐lung assessment of regional lung ventilation and perfusion through scanner hardware and acquisition improvements that can be adopted with relative technical simplicity.

Lung‐representative phantoms based on aqueous foams provided a valuable physical model to probe the impact of altering aqueous component magnetic susceptibility on the $T_{2}^{*}$ of PFP within the gas phase of the foam. While our foam bubble diameters were smaller than typical human alveoli (~200 m^35^), good agreement was observed between the theoretical and measured relationship between GBCA content and PFP $T_{2}^{*}$.

GBCAs are an essential tool in diagnostic MRI^36^ but have been associated with side effects such as the development of nephrogenic systemic fibrosis and the potential for accumulation of gadolinium within the brain. Our heathy volunteer study employed a well‐established GBCA at a dose consistent with current clinical guidelines.^37^ By comparison, our preclinical study used GBCA doses that were considerably higher than those used in our human experiments. This reflects the acquisition of preclinical data following equilibration of contrast agent through the mouse circulation (rather than employing dynamic visualization of contrast agent bolus passage through the pulmonary circulation) and an experimental design to observe complete matching of gas and aqueous component magnetic susceptibilities. It is quite feasible that a lower GBCA dose could be employed in future human ^19^F‐MRI DSC studies given the magnitude of change in $R_{2}^{*}$ observed. Furthermore, the use of GBCA in this setting can be considered in the context of current clinical methods to assess lung perfusion (namely, planar scintigraphy and computed tomographic pulmonary angiography) both of which incur an ionizing radiation dose.

## 5 CONCLUSION

DCE ^1^H‐MRI has previously been used to report on lung perfusion defects,^38^, ^39^, ^40^ employing T_1_‐weighted imaging to monitor GBCA bolus passage through the pulmonary vasculature. The DSC ^19^F‐MRI approach we present here differs from DCE ^1^H‐MRI in its ability to report on gas phase PFP directly adjacent to contrast agent that is present within the alveolar microvasculature. This holds potential for assessing lung perfusion at the alveolar level, applicable to basic physiology studies concerning gas exchange, with scope to develop for use in clinical populations (e.g., assessing the suitability for, and response to, long‐term oxygen therapy in patients with emphysema). Moreover, the approach is well suited to truly simultaneous assessment of alveolar ventilation and perfusion properties. Combined assessment of pulmonary ventilation and perfusion has previously been reported with ^19^F‐MRI of inhaled sulfur hexafluoride,^41^ exploiting the accumulation of SF_6_ in regions of low ventilation/perfusion ratio (V_A_/Q), coupled with the ability to measure SF_6_ partial pressure (sensitive to V_A_/Q) by change to its T_1_. Tracer‐free methods such as Fourier decomposition offer significant scope and appeal for assessment of lung function, enabling an indirect but entirely noninvasive approach to gauge ventilation and perfusion properties over the course of several respiratory cycles.^33^, ^42^, ^43^ Nonetheless, the DSC ^19^F‐MRI method reported here provides a rapid (single breath‐hold) approach that can be readily incorporated into existing ^19^F‐MR ventilation imaging. By its nature, DSC ^19^F‐MRI of inhaled PFP will only permit assessment of lung perfusion in regions of the lung that are adequately ventilated; yet, this provides an opportunity to report specifically on those regions of the lung that are directly participating in gas exchange (i.e., are both ventilated and perfused), which may be of wider clinical significance. Furthermore, studies employing dynamic imaging of inhaled PFP have previously demonstrated the ability for even poorly ventilated regions of the lung to exhibit gas wash‐in, albeit at slower rates than well‐ventilated regions.^8^ Thus, with appropriate experimental design, this limitation may be confined to regions of the lung where ventilation is truly absent (e.g., complete airway obstruction secondary to intrapulmonary mass). Further evaluation of this technique against existing MRI methods, as well as conventional pulmonary function tests, will be critical in assessing its potential to provide additional functional information relating to alveolar pathophysiology.
